# Tungsten Carbide Nanolayer Formation by Ion Beam Mixing with Argon and Xenon Ions for Applications as Protective Coatings

**DOI:** 10.1021/acsanm.2c05505

**Published:** 2023-02-22

**Authors:** Adel Sarolta Racz, Peter Kun, Zsolt Kerner, Zsolt Fogarassy, Miklos Menyhard

**Affiliations:** † Institute for Technical Physics and Materials Science, Centre for Energy Research, Konkoly Thege M. út 29-33, H-1121 Budapest, Hungary; ‡ Centre for Energy Research, Konkoly Thege M. út 29-33, H-1121 Budapest, Hungary

**Keywords:** tungsten carbide, nanocoating, ion irradiation, atomic force microscopy, scratch, hardness

## Abstract

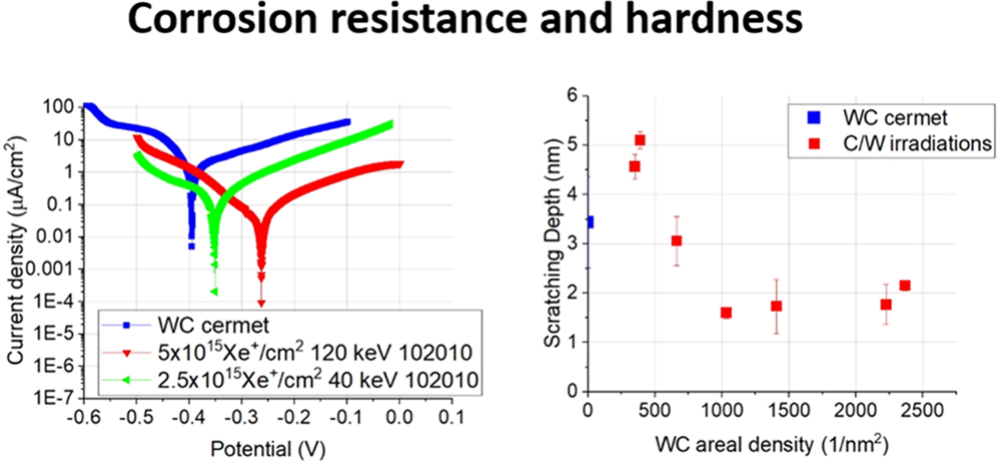

A novel nanolayer is formed by means of ion irradiation applicable as protective coating. Tungsten carbide (WC)-rich nanolayers were produced at room temperature by applying ion beam mixing of various carbon/tungsten (C/W) multilayer structures using argon and xenon ions with energy in the range of 40–120 keV and fluences between 0.25 and 3 × 10^16^ ions/cm^2^. The hardness of the nanolayers was estimated by means of standard scratch test applying an atomic force microscope equipped with a diamond-coated tip (radius < 10 nm); the applied load was 2 μN. The irradiation-induced hardness of the nanolayers correlated with the areal density of the WC; with the increasing amount of WC, the hardness of the nanolayer increased. The produced layers had an order of magnitude better corrosion resistance than a commercially available WC cermet circular saw. If the WC amount was high enough, the hardness of the layer became higher than that of the investigated WC cermet. These findings allow us to tune and design the mechanical and chemical properties of the WC protective coatings.

Durable materials serving as protective coating in harsh environmental conditions are essentially important in various applications, eg., sensors, medical instruments, and new energy generating systems. Candidate materials with superior mechanical durability and corrosion resistance include nitrides (TiN and Si_3_N_4_) and carbides (SiC, BC, TiC, ZrC, and WC).^1−4^ Tungsten carbide is a good choice as a protective coating, since it has a high melting point (a peritectic melting temperature of 2785 °C), hardness, and corrosion resistance. A variety of techniques are available to produce WC films, like physical vapor deposition (PVD) and atmospheric-pressure chemical vapor deposition (CVD). PVD processes can leave high residual stress in the material, while in the case of CVD, the application of hazardous gases and elevated temperatures might be disadvantageous for certain applications.^5,6^

WC is also widely used as cermet; in this case, it involves the formation of a composite with a binder metal, such as Co or Ni. The corrosion resistance of cemented carbides is generally modest because the binder and efforts to find proper cheap binders are important.^1,7−9^ Furthermore it has been shown that the WC and Co mixture is toxic.^10^ This is not true for the individual WC, for instance, a novel zinc/tungsten carbide nanocomposite has been produced as a bioabsorbable implant.^11^ Another example is the production of a WC-based microbial fuel cell.^12^ Binderless WC production by a nontoxic method at room temperature can be an evident solution for the above problems.

Ion beam irradiation can be used to produce coatings. It is a nonequilibrium process, which might cause severe changes of the material as various defect formation, intermixing, compound formation, metastable phase formation, hardening of material, etc., which are mostly detrimental but sometimes beneficial processes. Tremendous amount of work is known for high ion and neutron energy cases. It was shown that, due to high energy irradiation, precipitation and compound formation occurred.^13,14^ Similarly, many studies deal with the hardening of the material mainly for the safety reasons of the nuclear power stations.^15−17^ Similar processes are active at lower ion energies (0.1 MeV), but obviously the thickness of affected region is only in the range of a few tens of nanometers, which is usually unimportant for macroscopic cases but especially important for nanolayers. Milosavljevi′c et al. applied medium-energy (180 keV Ar^+^) ion irradiation on a Ni/Ti multilayer system to produce a thin Ni–Ti amorphous layer, which is an important system in optics and actuators.^14^ Racz et al. applied also medium-energy (40–120 keV) ion irradiation for producing a SiC nanolayer.^18−20^ Zhang et al. used 200 keV Xe^+^ ions for the production of a metastable C–W phase.^21^ Wang et al. bombarded a 100-nm-thick carbon layer deposited on tungsten by 40 keV argon or nitrogen ions and detected WC at the interface.^22^ In the above works, the focus was rather on the interface reactions not on the characterization of the chemical and mechanical resistance.

Previously we have shown that ion beam irradiation causes intermixing of the W/C multilayer, resulting in a WC-rich nanolayer at room temperature.^23^ The corrosion resistance of the nanolayer was excellent and thus considerably better than that of a WC cermet.^24^

Herein, we report on the mechanical feature, hardness, of the WC-rich nanolayer produced by ion beam mixing. The scratching test was performed on an atomic force microscope (AFM), equipped with a diamond-tipped cantilever. The hardness of the samples was estimated from the scratch test experiment. It turned out that the hardness increased with the increasing amount of WC, which increases with fluence, that is, irradiation-induced hardening was observed. The highest hardness of the WC-rich nanolayer exceeded the estimated hardness of the WC cermet. These findings show that these protective coatings have high hardness and are highly corrosion-resistant.

## 2 Experimental Section

### 2.1 Production of WC-Rich Nanolayers

The WC-rich nanolayers were produced by means of ion beam mixing (IBM) of different C/W multilayer structures.

The initial C/W multilayer structures were made on a Si single crystal by sputter deposition of pure C and W layers; the detailed description of the procedure is given in ref (25) Its essence is the following. The sputtering was made in a Balzers Sputron sputtering chamber in the Jožef Stefan Institute, Ljubljana. The sample was far from the plasma; thus, during the layer growth, the temperature was always below 100 °C. The thickness of the sputtered layer was controlled by quartz-crystal microbalance. The following structures have been made for this study: C (10.4 nm)/W (24.5 nm)/C (9.2 nm)//Si substrate; C (8 nm)/W (18nm)/C (8.7 nm)/W (18.6 nm)/C (7.1 nm)//Si substrate; C (15.8 nm)/W (22.7 nm)/C (17.2 nm)/W (24.3 nm)/C (21.1 nm)//Si substrate; for easier reference, we call these samples 102010, 1020, and 2020.

For the ion beam mixing, we applied argon (40–110 keV, the fluences 0.1–6 × 10^15^ Ar^+^/cm^2^) and xenon ions (40–160 keV, fluence 0.07–5 × 10^15^ Xe^+^/cm^2)^ at room temperature (Helmholtz Zentrum Rossendorf Dresden in a High-Voltage Engineering Europa B.V., Model B8385 implanter).

For a comparison, the properties of a commercially available WC cermet were also measured. A circular saw was purchased from Mecut, Ceranisi, Italy, consisting of a WC–Co layer prepared by powder metallurgy. Before the measurements, it was polished achieving a roughness below 7 nm.

### 2.2 AES Analysis

AES depth profiling was applied to reveal the concentrations of elements and compounds along the depth after the various ion bombardments. The detailed description of the AES depth profiling is described elsewhere.^18^ Summarizing, the measured C (KLL) Auger peak could be decomposed into graphitic and carbide components (see Figure 2 in ref (25)). The relative sensitivity factor method for the calculation of the atomic concentrations has been applied.^26^ The sputtering time was transformed to removed thickness for getting depth profiles.^27^ Hence, the AES analysis provided the in-depth distributions of Si, C, WC, and Ar or Xe. The only difference from the usual arrangement was that the angle of incidence of the ion bombardment was chosen to be 65° with respect to the surface normal; this unfavorable angle of incidence was chosen to cope with the large difference of the sputtering yields of W and C.^28^

### 2.3 XTEM Studies

The structure of the pristine and irradiated specimens was determined by cross-sectional transmission electron microscopy (XTEM). The XTEM measurements were performed in an FEI-Themis Cs-corrected (scanning) transmission electron microscope, in both high-resolution electron microscopy (HREM) and scanning transmission electron microscopy (STEM) mode (point resolution is around 0.09 nm in HRTEM mode and 0.16 nm in STEM mode) operated at 200 kV. The sample preparation for XTEM was made by FIB ion-milling.

### 2.4 AFM Studies

Both the surface topography and the scratch resistance was investigated by a Bruker Multimode 8 AFM equipped with a closed-loop scanner applying a single-crystal diamond tip (SCD ART D300 probes, tip radius <10 nm, nominal spring constant 40 N/m, resonant frequency 300 kHz) mounted on a stainless steel cantilever. Due to the high sensitivity and small tip radius, the diamond tip was able to obtain surface morphology and to create constant-force scratches in the surface. The surface topography was determined in tapping mode.

#### 2.4.1 Scratch Test

The AES depth profiles have shown that, in the majority of the cases, not the whole upper carbon layer was consumed by the IBM-induced compound formation. Therefore, the WC-rich region produced was covered by the remaining carbon (not used up by the IBM) layer of various thicknesses; the thickness decreased with increasing fluence of the IBM. To be able to determine the mechanical feature of the WC-rich region, the remaining pure C layer has been removed by oxidation in microwave plasma. This procedure removed the graphitic C but did not affect the WC-rich region.^18,24^

**Figure 1:**
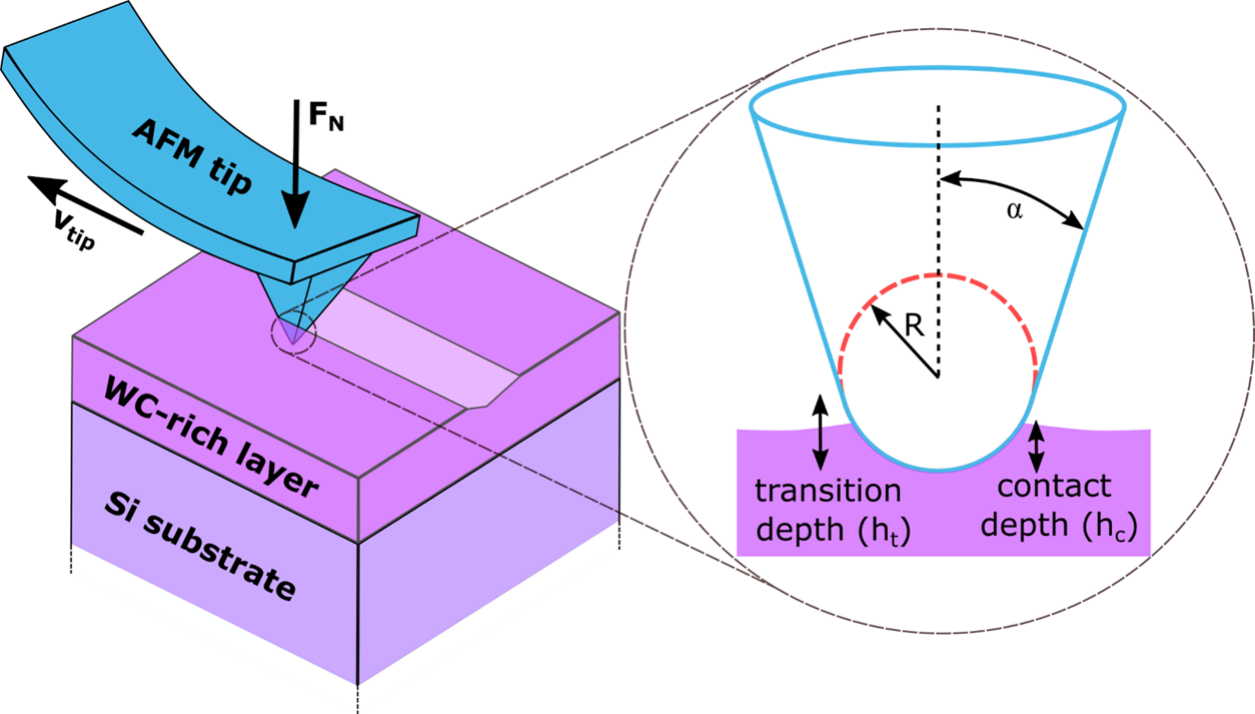
Scheme of the scratching test performed by AFM together with the projected contact area.

Each sample received parallel scratches with a normal force of 2 μN. The applied loading force was derived from the bending of the actual cantilever and its spring constant measured by thermal noise. The scratching has been controlled by the NanoMan lithography software of Bruker. The following procedure had been applied: (a) imaging of the area, (b) creation of 1 μm long scratches with the preset force, the sliding speed was 800 nm/sec, and (c) imaging of the area of the scratches with the same tip. The images were evaluated by applying Gwyddion^29^ software. The scratch depth was calculated over several scan lines and averaged. For better understanding, a scheme of the scratching test procedure together with the projected contact area is provided in Figure 1.

### 2.5 Measurement of the Corrosion Resistance

This topic is discussed in a recent publication.^24^ Summarizing, potentiodynamic corrosion tests were performed with a computer-controlled Gamry Reference 3000 potentiostat in a three-electrode glass cell in deaerated 3.5 w/w% NaCl solution. The reference was a saturated calomel electrode (SCE), which was immersed with the help of a Luggin capillary. A platinum mesh was applied as the counter electrode. The working electrode (contact area 0.3 cm^2^) was contacted with the solution by applying the hanging meniscus technique. The curves were evaluated by the Tafel extrapolation method.

**Figure 2:**
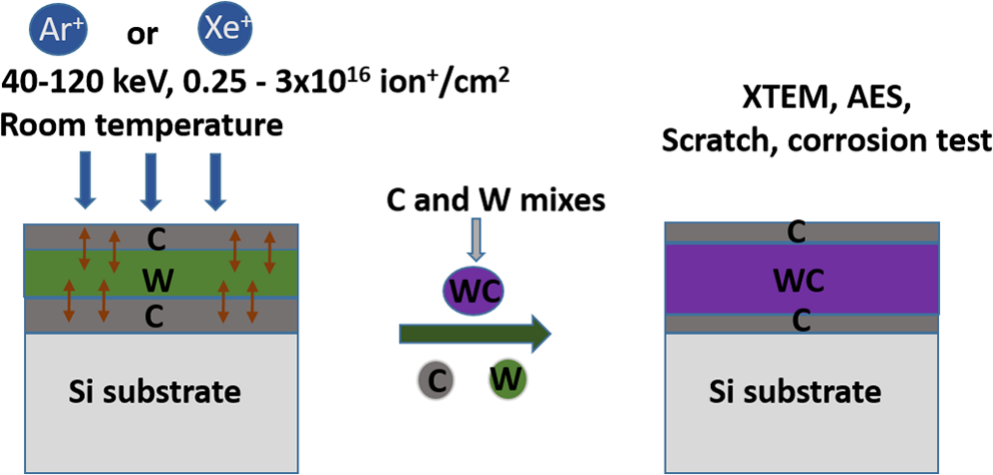
Scheme of the WC-rich layer fabrication method.

Summarizing the experimental part, a scheme of the WC-rich layer fabrication method is provided in Figure 2 for better understanding.

## 3 Results and Discussion

**Figure 3:**
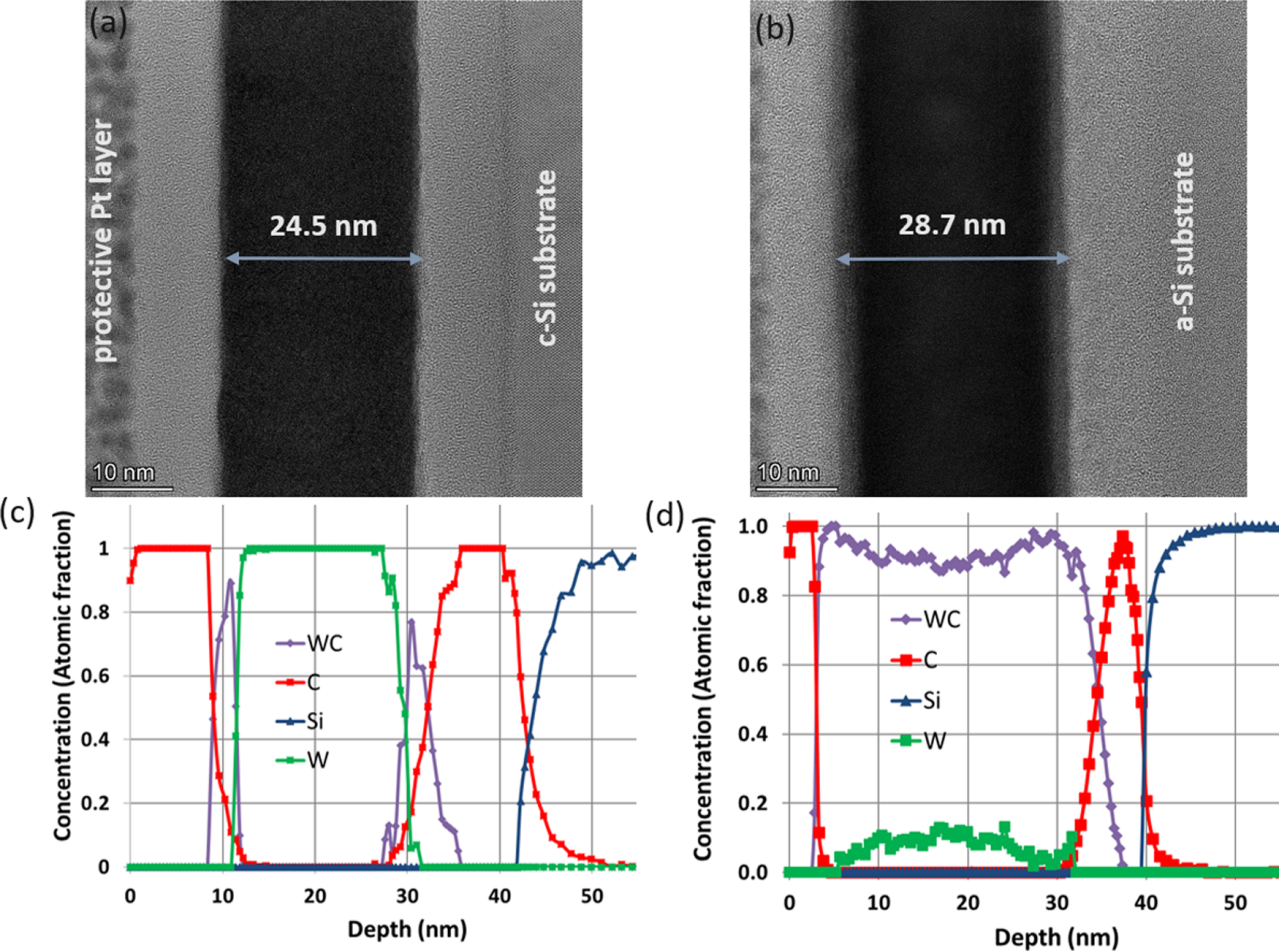
HRTEM image of the (a) 102010 pristine and (b) 120 keV, 5 × 10^15^ Xe^+^/cm^2^ 102010 irradiation and the corresponding as-measured AES depth profiles (c, d) of the samples.

The HRTEM XTEM image of a 102010 pristine and 5 × 10^15^ Xe^+^/cm^2^ irradiation is shown in Figure 3a,b. In the pristine (a) sample, all interfaces are sharp, and we can see three amorphous layers, which are deposited on a crystalline substrate. The darker region corresponds to tungsten, the material of higher atomic number. The HRTEM image of the irradiated sample (b) shows that serious changes happened in the sample. The substrate is partially amorphized, and the thickness of the carbon layers is strongly reduced. Still, the interfaces are stripes parallel to the surface, that is, the planar architecture of the sample has been preserved despite the strong material transport. The samples remained amorphous after the ion irradiation. The WC compound formation can be detected by AES where the carbon Auger peak is very sensitive to the change of the bonding state. The corresponding as-measured AES depth profiles (Figure 3c,d) show the detailed composition of the layers. In the pristine sample (Figure 3c), we can see that the interfaces are not sharp and thin WC layers appear at the interfaces; this is an artifact caused by the argon sputtering applied for AES depth profiling.^25^ Having corrected the measured depth profiles for the artifact results in pure C/W/C layers with sharp interfaces according to the XTEM image.^25^Figure 3d clearly shows the serious changes due to ion irradiation. A continuous WC layer was produced by IBM (here, the artifact results an about 10% increase of the thickness of the WC layer^25^) of W and C atoms and the concomitant compound formation. Obviously a large part of the C has been used for the WC layer, and thus, the thicknesses of the carbon layers are decreased.

**Figure 4:**
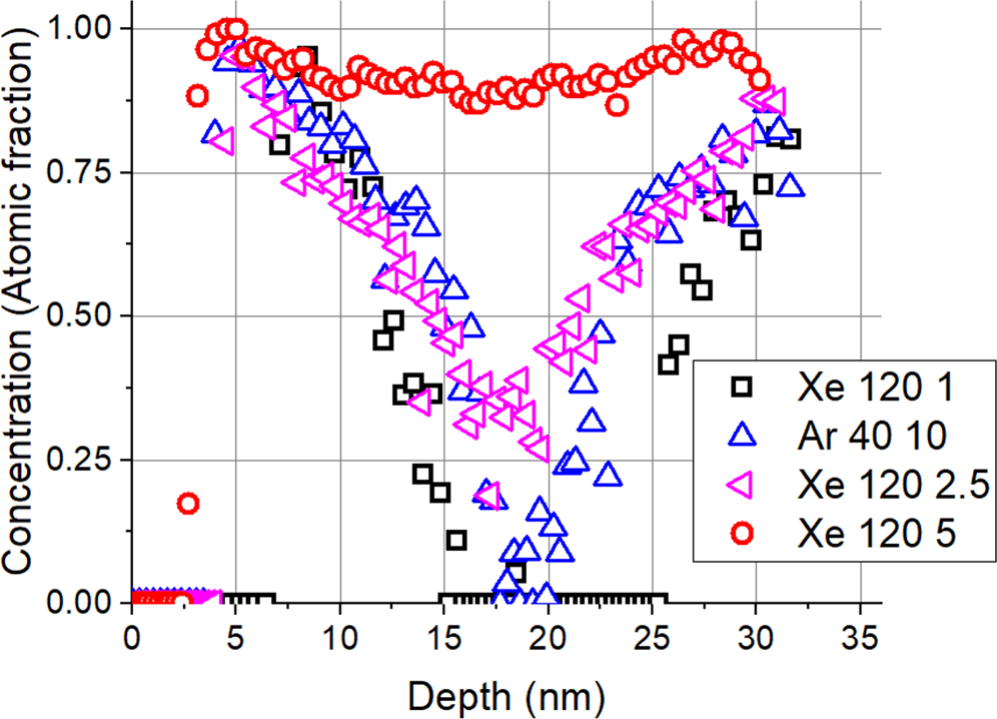
Typical WC distributions measured by AES on sample 102010. The legends give the projectile, the energy (unit keV) of the projectile, and the applied fluence (unit 1 × 10^15^/cm^2^).

Depending on the conditions of the ion irradiation and sample structure, various WC distributions are produced as it is discussed in recent papers.^23,24^ Some typical AES in-depth distributions are shown in Figure 4.

These examples show that the IBM-induced WC formation starts at the interfaces, and with increasing fluence, these originally separated WC-rich regions merge and finally a well-developed layer forms, allowing to study the chemical and physical behavior of various WC-rich nanolayers.

**Figure 5:**
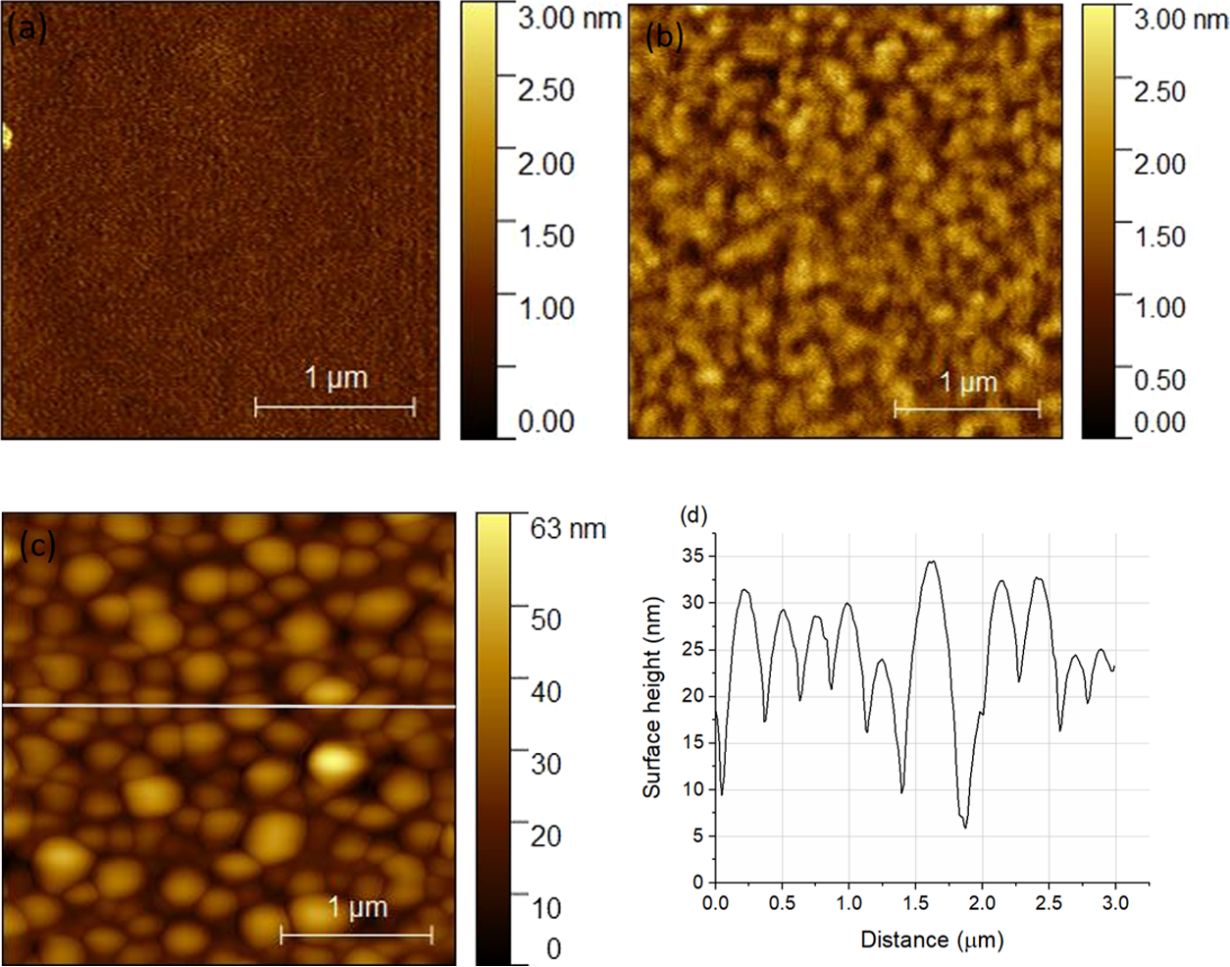
Typical surface morphologies of (a) 102010 pristine, (b) 102010 5 × 10^15^ Xe^+^/cm^2^, 120 keV irradiated, and (c) 3 × 10^16^ Ar^+^/cm^2^, 40 keV irradiated with (d) corresponding line profile samples.

Figure 5a shows the AFM topography image of a 102010 pristine sample; it can be seen that the surface is smooth, and the root-mean-square roughness (RMS) was 176 pm. Figure 5b,c shows two typical morphologies after the ion irradiation; the 102010 sample has been irradiated by 5 × 10^15^ Xe^+^/cm^2^, 120 keV and 3 × 10^16^ Ar^+^/cm^2^, 40 keV, respectively.

Figure 5b shows that, comparing to the pristine sample, some roughening occurred, but the surface is still smooth, the RMS being 320 pm. Figure 5c (a high irradiation of 3 × 10^16^ Ar^+^/cm^2^, 40 keV) presents a strongly different morphology together with a corresponding line scan (Figure 5d). It can be seen that specific features appeared on the surface with a height of 9–24 nm and width of 220–500 nm. This morphology development is explained as the result of Ar bubble formation. These features prevent the meaningful application and interpretation of scratch tests on such surfaces. Thus, before going into the details of the scratching tests, we must describe the morphology development due to ion irradiation.

Considering the angle and energy (0° with respect the surface normal and 40–160 keV) of the irradiating ions, backscattering is negligible, and their penetration is in the range of 20–100 nm. Some of them can escape from the sample by means of the accelerated diffusion due to the strongly excited condition of the bombarded matrix. The majority of the entered atoms, however, remain in the sample, originally mainly in interstitial places. Again considering the strong atomic movements during ion irradiation, the possibility of reaching lower energy conditions is relatively high. The energy of the system if the projectiles leave the interstitial position and form bubbles decreases; this is a well-known process.^30−33^ We assume that the surface morphology development is mainly connected to the bubble formation. Since the bubble formation depends on the amount of projectiles, its concentration is to be known for planning the experiments.

**Figure 6:**
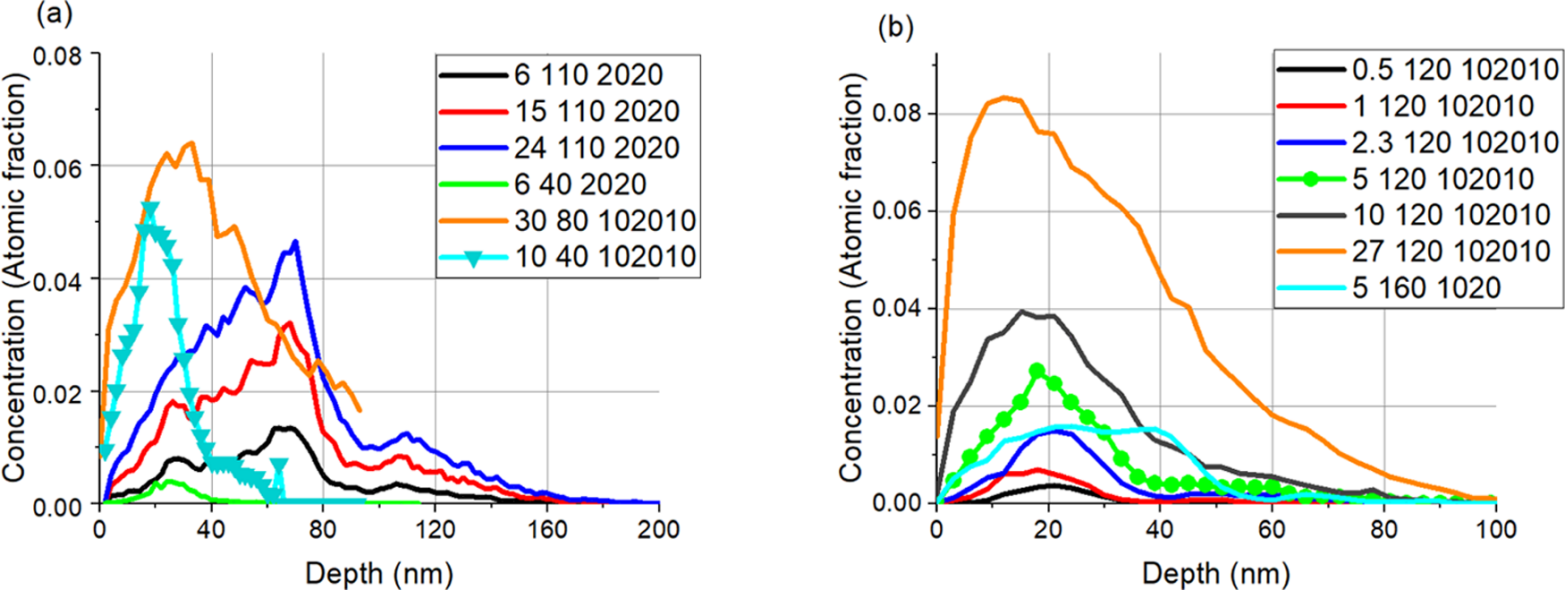
Simulated (a) Ar^+^ and (b) Xe^+^ distributions. The numbers in the legend stand for irradiation fluence (×10^15^/cm^2^), energy (keV), and sample type. The curve signed with symbols shows the limit for morphology development; fluence providing lower and higher projectile concentrations resulted in serious morphology development.

The concentration distribution of the projectiles has been measured by AES; however, due to the low concentrations and relative sensitivity factors of Xe and Ar, the measured Auger signal is rather noisy. On the other hand, we checked if the TRIDYN simulation^34^ correctly describes it. It turned out that the agreement between the simulation and measurement is reasonably good.^23^ Thus, instead of the measured projectile distributions, the simulated ones will be used for the determination of the amount of projectiles. Figure 6 a,b shows several distributions of Xe^+^ and Ar^+^ projectiles in various samples after various irradiations, which was compared with the available AFM images. It turned out that one can easily find a critical projectile concentration distribution, which shows the onset of the serious morphology development.

Figure 6 shows that the morphology development depends on the projectile as well; in the case of Xe^+^ irradiation, the morphology development starts at somewhat lower fluence than that in the case of Ar^+^ irradiation, and based on the TRIDYN simulation, one can easily find irradiation conditions, which do not result in serious morphology development. For all scratching tests, irradiations not producing serious morphology development were chosen, that is, all scratching tests were performed on smooth surfaces.

**Figure 7:**
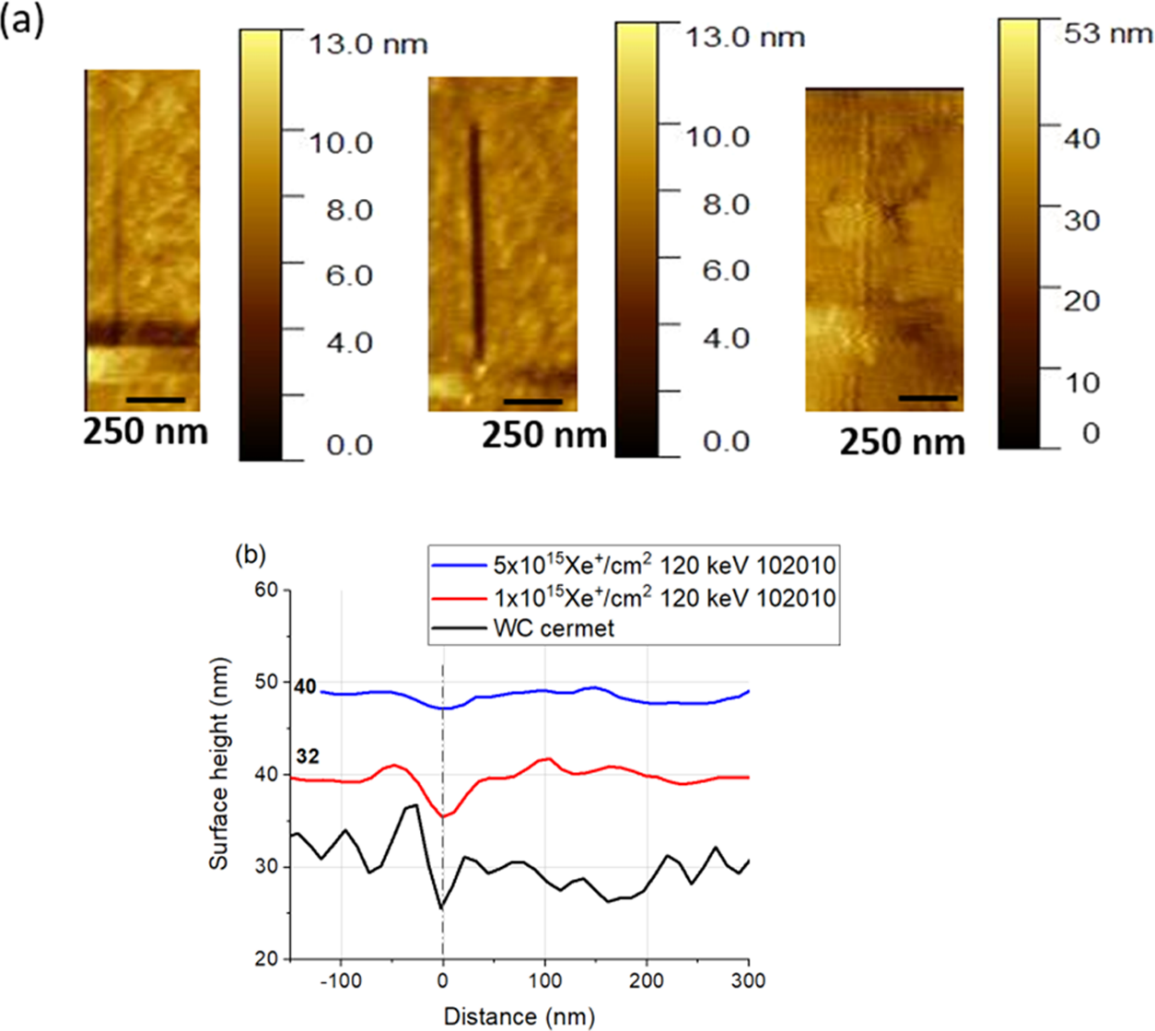
Results from scratch testing of 1 × 10^15^ Xe^+^ /cm^2^ 120 keV, 5 × 10^15^ Xe^+^/cm^2^ 120 keV (102010), and WC–Co cermet by an AFM diamond tip with a radius of less than 10 nm. (a) AFM image of the scratches formed under a load of 2 μN. (b) Corresponding line scan of the scratches. For better visibility, the curves are shifted, by 32 and 40 nm as indicated in the figure.

As an example, the AFM images of the scratches made on irradiated samples together with a commercially available WC–Co cermet, as a comparison, are shown in Figure 7.

Figure 7 emphasizes that the scratching tests were performed on smooth surfaces, and the depth of the scratched lines seems to depend on the sample, that is, on the WC amount and distribution. It is also shown that, in the case of higher fluence irradiation, the scratch depth becomes lower than that of the WC–Co cermet.

**Figure 8:**
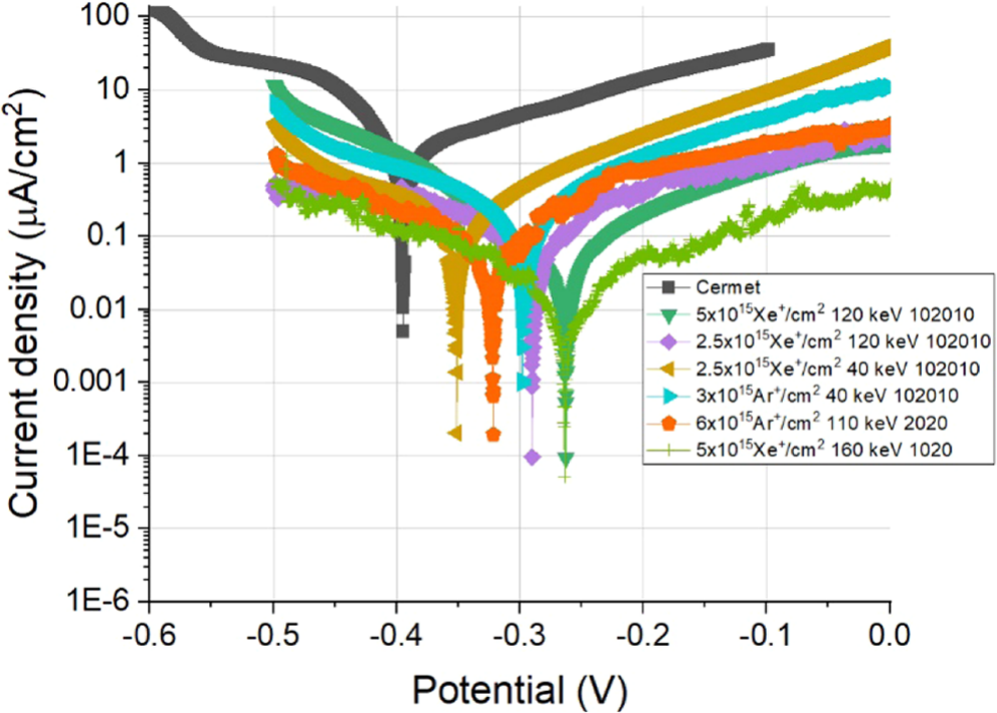
Polarization curves obtained from samples on which successful scratching tests were performed.

Figure 8 shows the results of the corrosion test performed on samples providing the successful scratching test. The Tafel curves show that the irradiated samples have lower current densities and more positive potential than the WC cermet, proving the corrosion resistive aspect of the samples. This high protectivity is due to the absence of the corrosive Co binder. The corrosion current density data available in the literature are in the range of some tens of μA/cm^2^ but vary greatly for WC–Co cermets due to the difference of different production and measurement methods.^35−37^

It can be also seen that the irradiated samples differ in the current densities showing the different protectivity of the irradiated probes. Previously we have studied the corrosion resistance of WC-rich layers produced by ion beam mixing.^24^ In that case, it had been shown that the corrosion resistance does not depend linearly on the amount of WC, rather the corrosion resistances of the samples are poor and excellent under and above a certain WC amount (distribution), respectively.^24^ Considering Figure 4, in the case of 1 and 2.5 × 10^15^ Xe^+^/cm^2^, 120 keV irradiations for sample 102010, the corrosion resistance is poor, while larger 5 and 10 ×10^15^ Xe^+^/cm^2^, 120 keV irradiations produce samples with excellent corrosion resistance.^24^ This behavior could be described by introducing the quantity of effective areal density,^18^ which was successfully applied in the similar case of SiC-rich layers also produced by IBM. The simple meaning of this description is that, to have a good corrosion resistance, one needs a coherent WC layer. Now, it is a question whether in the case of scratch test, the same evaluation routine works.

For the characterization of the mechanical behavior of thin layers, generally nanoindentation is used, which provides the hardness and yield strength of the layer, which can be compared with those of the bulk material.^38^ Nanoindentation can only be used if the total thickness of the material to be measured is large enough; the indentation depth cannot be larger than 10% of the total thickness. In the case of nanolayers, this condition usually cannot be met since it results in unphysically low indentation depths. Instead of nanoindentation, it is more common to use scratch tests.^39−42^ It was invented long time ago for comparing the hardness of bulk minerals.^43,44^ The scratching test measures the resistance of material against plastic plowing, and a series of measurements can be used to exhibit the relative strengths of the materials involved (like for the case of original proposal). Still, to appreciate the results obtained, it is good to compare them to some macroscopic measure. Since the nanoindentation, from which the macroscopic hardness can be calculated, also probes the plastic behavior of the surface close region of the material, there is hope that the two methods could be compared.^42^ Because of the many parameters involved, the exact connection between scratch hardness and indentation hardness is not known, but monotonic relation exists. Thus, using several assumptions, we will estimate the macroscopic hardness of our nanolayers.

The scratch hardness *H*_S_ is defined as the link between normal force and projected contact area *A*_S_ during steady-state scratching^42,45^
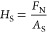
1For calculating the projected contact area (see the sketch in Figure 1), the three-sided pyramidal AFM tip is considered as pyramid with a spherical crown at the cusp of the tip.^46^ It is assumed that, only the front half of the tip is in contact with the material, thereby forming a half-circular projected contact area. There are two expressions for the contact area for sphero-conical tips during scratching.^28^ At the transition depth *h*_*t*_, the spherical part transitions into the conical part
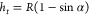
2Here, *R* is the radius of the tip-sphere and α is the half-angle of the conical part. Calculating *h*_*t*_ with the data provided by the tip manufacturer, the result is around 7 nm. In our case, the contact depth (*h*_c_) was always below the transition depth; therefore, the area function can be considered as
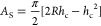
3

**Figure 9:**
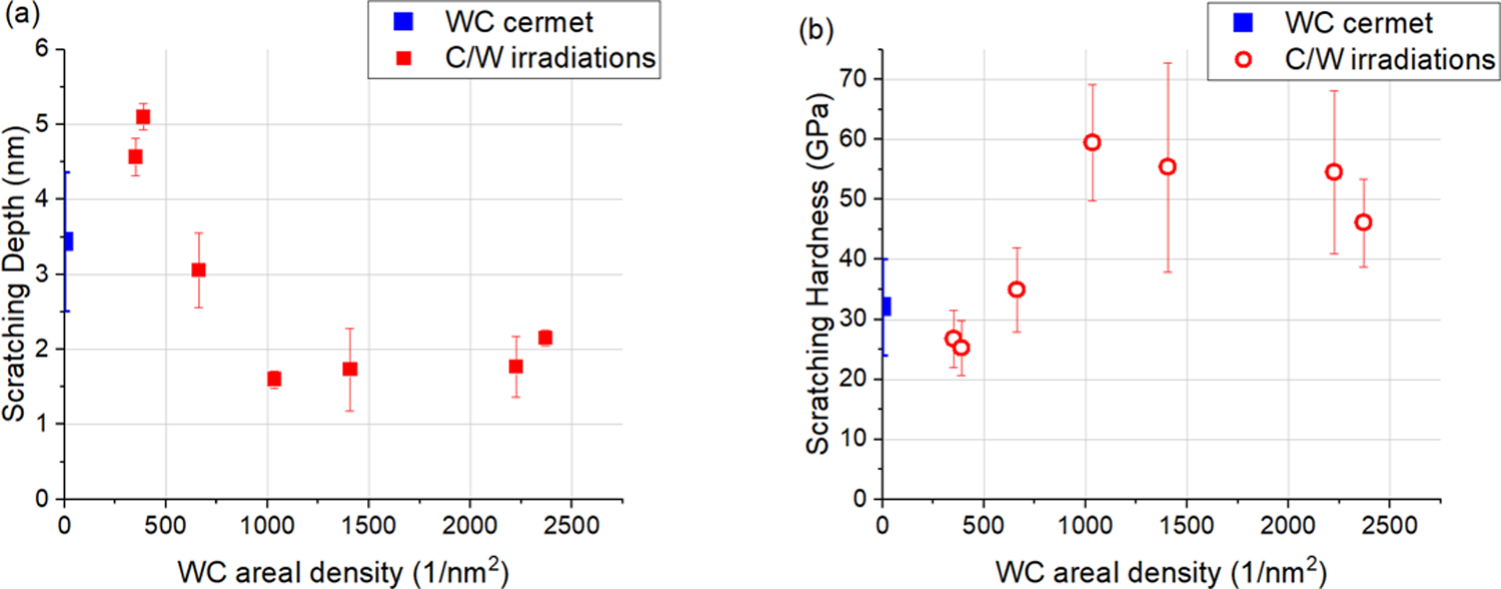
Scratching (a) depth and (b) estimated scratching hardness vs. areal density. The value at zero areal density refers to the scratching depth measured on cemented WC.

Experiments clearly showed that the measured characteristic features, corrosion resistance and hardness, of the irradiated samples depended on the fluence, which resulted in various WC distributions among others. Our assumption is that the appearance of the WC causes the observed changes of the corrosion resistance and hardness. Considering Figure 4, which shows various possible WC distribution, it is far not trivial how we can characterize them. It turned out that, in the case of the scratch test, the same evaluation routine (WC measured by effective areal density) that worked in the case of corrosion test did not work. Therefore, we chose to check the dependence of the scratching depth on the areal density of WC; the areal density of an element and/or compound is its integral along the depth, that is, the areal density gives the whole amount of element and/or compound in the material. Note that the thickness of the whole region containing WC is in the range of some tens of nanometers. Figure 9 summarizes scratching depth values and corresponding estimated scratching hardness values of all studied samples (for details, see Table 1), as a function of the areal density of WC.

**Table 1:**
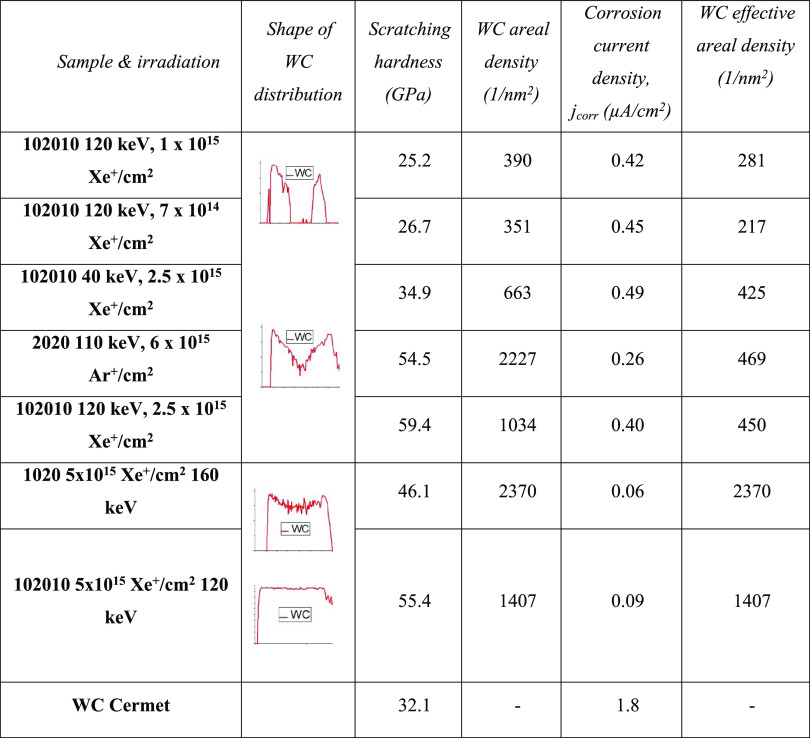
Scratching Hardness (Third Column) and Corrosion Current Densities (Fifth Column) Together with the Areal (Fourth Column) and Effective Areal (Sixth Column) Densities for Samples with Given Irradiation (First Column) and WC Distribution (Second Column)

For comparison, the scratching depth measured on cemented WC–Co applying the same conditions is also shown in Figure 9 at the zero areal density point.

Figure 9a shows that the scratching depth decreases as the areal density of the WC increases. Similarly, the scratching hardness of the layers, estimated from the scratching depth, Figure 9b, depends on the areal density of the WC. Figure 9b clearly shows that the scratching hardness of the irradiated material increases with increasing fluence; irradiation-induced hardening occurs. It is also clear that, upon reaching a given value of WC amount, the hardness saturates. It should be emphasized that Figure 9 contains only measurements where the bubble formation-induced morphology development is weak.

Table 1 summarizes all experimental results (corrosion and scratch test) including the preparation of the samples and sketch of the WC distributions necessary for the calculation of the areal and effective areal density.

Table 1 clearly shows the strongly different dependences of the scratching hardness and corrosion resistance on the fluence, that is, on the amount of WC produced. While the hardness quasi-monotonically increases with the amount of WC particles, the corrosion resistance is more or less constant before and after the formation of a continuous WC layer.^24^

It can be concluded that, while the corrosion resistance depended on the effective areal density, the hardness depends on the areal density. This also means that the mechanisms of the two processes are strongly different. In the case of the scratching test, it seems that even the individual small WC particles contribute to the resulting hardness; this behavior is similar to that of precipitation-hardened material.^13,47^ With the increase of the number of the WC particles, the scratching depth decreases, that is, the hardness of the layer increases. The saturation value of the scratching depth is lower than that of the cemented WC, that is, our nanolayer exceeded the hardness of the macroscopic cemented WC chosen for comparison. It should be added that reported hardness values measured by nanoindentation on WC–Co cermets varies in a wide range of 13–79 GPa;^48,49^ therefore, our hardness value measured by the scratch test falls in the literature range. It is also worthy to note that the measured scratch hardness can be larger than the bulk value^50^ due to the submicrometric penetration depths.

Unfortunately, we could not follow the study of this process for a longer period (higher fluences) because of the appearing of the undesired surface morphology. From the point of view of the nanolayer quality, it should be emphasized that though in a limited fluence region, there are nanolayers of which both the scratching depth and corrosion current density are low. It means that we could produce layers exhibiting good hardness and corrosion resistance reaching the goal of our study. This condition is reached when the effective areal density equals with that of the areal density, that is, when the coherent WC layer forms.

## 4 Conclusions

Various C/W multilayer structures were ion-irradiated with various projectiles and energies to a fluence not causing accelerated morphology development on the surface. The scratching depth was measured by applying an atomic force microscope equipped with a diamond-tipped cantilever. The scratching hardness of the nanolayer was estimated from the scratching depth, which depended on the areal density of WC. With increasing fluence, the areal density of WC increases as well as hardness, showing that irradiation-induced hardening occurs. The hardness reaches a saturation value better than that of the studied WC cermet. The corrosion resistance of the nanolayer follows another dependence; it depends on the effective areal density. At irradiations, when the areal density agrees the effective areal density, both the hardness and corrosion resistance are excellent. Thus, we could produce nanolayers applicable as protective coating with high hardness and corrosion resistance.
